# Association between maternal PAH exposure and immune-inflammatory indices during pregnancy

**DOI:** 10.3389/fimmu.2026.1852582

**Published:** 2026-06-09

**Authors:** Lin Tao, Yi Zhang, Yuan-zhong Zhou, Xubo Shen

**Affiliations:** 1 School of Public Health, Zunyi Medical University, Zunyi, Guizhou, China; 2 Centre for Disease Control and Prevention, Guiyang, Guizhou, China; 3 The People’s Hospital of Qian Nan Prefecture, Qian Nan, Guizhou, China

**Keywords:** birth cohort study, maternal immune-inflammatory indices, polycyclic aromatic hydrocarbons, pregnancy, systemic inflammation

## Abstract

**Introduction:**

Polycyclic aromatic hydrocarbons (PAHs) are endocrine-disrupting chemicals, yet the relationship between maternal PAH exposure during pregnancy and maternal immune-inflammatory indices remains poorly understood. Therefore, we included 2581 pregnant women in the Zunyi birth cohort and measured their concentrations of PAH metabolites during pregnancy and collected pregnancy related immune examination indicators to explore the association between PAH metabolites in pregnancy urine and maternal immune inflammation indicators.

**Methods:**

Generalized linear regression models were used to assess correlations between PAH metabolites and immune-inflammatory indices. Restricted cubic spline (RCS) models were employed to characterize dose-response relationships. Bayesian kernel machine regression (BKMR) models evaluated overall effects and non-linear associations of PAH metabolites with these indices. Weighted quantile sum (WQS) regression quantified the relative contribution of individual PAH metabolites to immune-inflammatory alterations.

**Results:**

Our results showed that 1-Hydroxynaphthalene (1-OHNAP), 2-hydroxynaphthalene (2-OHNAP), 2-hydroxyfluorene (2-OHFLU), and 9-hydroxyphenanthrene (9-OHPH) are negatively correlated with white blood cell (WBC) count, positively correlated with systemic immune inflammation index (SII), negatively correlated with systemic inflammatory aggregation index (AISI), negatively correlated with neutrophil to lymphocyte ratio (NLR), and positively correlated with platelet to lymphocyte ratio (MLR). Additionally, RCS models identified dose-response relationships between 1-OHNAP, 2-OHNAP, 1-hydroxypyrene (1-OHPYR), 9-OHPH, 2-hydroxyphenanthrene (2-OHPH), and 4-hydroxyphenanthrene (4-OHPH) and the aforementioned indices (excluding PLR). Furthermore, BKMR models demonstrated that PAH metabolites were negatively associated with WBC count and NLR; at low combined concentrations, they were negatively associated with MLR and positively associated with PLR, with no such associations observed for SII or AISI. WQS regression identified 9-hydroxyfluorene (9-OHFLU), 9-OHPH, 4-OHPH, 2-OHPH, and 1-hydroxyphenanthrene (1-OHPH) as key metabolites driving these associations.

**Conclusion:**

Collectively, our findings demonstrate that maternal exposure to PAH metabolites during pregnancy is associated with maternal immune-inflammatory indices. Limiting PAH exposure during gestation may help protect maternal and fetal health.

## Introduction

Polycyclic aromatic hydrocarbons (PAHs), compounds formed in incomplete combustion reactions and emitted mainly through air pollution and fuel combustion (1), have carcinogenic and endocrine-disrupting properties (2). PAH metabolites can enter the body through inhalation, ingestion, and dermal contact, and are excreted in urine and feces; therefore they tend to accumulate in the body over time. In addition, exposure to PAH has been identified as a potential health risk to pregnant women (3). In addition, exposure to PAHs has been identified as a potential health risk to pregnant women (3).

The immune system is intricate, and its dysregulation contributes to numerous diseases, including non-communicable disorders (4). Environmental pollutants (e.g., synthetic and natural chemicals) can drive immune dysregulation (5, 6). Pregnancy induces changes in the maternal immune system—particularly early gestation, coinciding with marked hormonal shifts—often accompanied by pregnancy-related reactions and reduced immune competence. Maternal immune cells are critical for establishing and maintaining pregnancy (7, 8) and modulate susceptibility to prenatal and pregnancy-related complications (9). Key immune cells include natural killer (NK) cells, macrophages, T cells, and dendritic cells (10, 11), which—beyond preventing fetal immune rejection—drive trophoblast invasion, tissue remodeling, and angiogenesis to form a healthy placenta (12, 13). Derived from hematopoietic stem cells (maturing in the fetal liver and bone marrow; 14, 15), immune cells mediate humoral and cellular immunity. Though low in abundance, they gain growing attention for supporting healthy pregnancy via antibody production and other cellular regulation (16). Immune cell proportions rise moderately between 27–33 weeks of gestation, then decline slightly at term (17). In mice, absent mature immune cells correlate with smaller litters and embryo sizes (18); in humans, reduced immune cell levels link to elevated risks of preterm birth (PTB), pre-eclampsia, recurrent miscarriage, stillbirth, and vaginal hemorrhage (19–21).

However, the link between prenatal maternal PHE exposure and circulating immune-inflammatory biomarkers remains unclear. Therefore, this study included 2581 pregnant women in the Zunyi birth cohort and used multiple statistical methods to assess the relationship between PAH exposure during pregnancy and maternal immune-inflammatory biomarkers. This will provide epidemiological evidence for the promotion of maternal and child health.

## Methods

### Study population

The study population was drawn from the Zunyi Birth Cohort (ZBC), a cohort study initiated in April 2020 and completed in May 2022. Inclusion criteria for the ZBC: age between 20 and 45 years, spontaneous conception, singleton pregnancy. Exclusion criteria: severe chronic and infectious diseases (e.g., cancer, diagnosed cardiovascular disease, chronic renal failure, and HIV infection). For more information about ZBC, please refer to previous studies (22, 23), which were approved by the Ethics Committee of Zunyi Medical College (No. [2019] H-005), and each pregnant woman who participated in the study provided informed consent. A total of 2581 pregnant women were included in this study, and all of them had a complete dataset available for analysis, including baseline information, urinary PAH exposure, and maternal immune cell levels. See **Supplementary Figure 1**.

### Sample collection & testing

The collection of urine covers the entire pregnancy period, and ten metabolites of polycyclic aromatic hydrocarbons in urine were measured by gas chromatography-mass spectrometry (GC-MS) and corrected for creatinine. For detailed methods, please refer to our previously published literature (22, 23). The quantification of PAHs in urine was performed using the standard internal standard method, as outlined in **Supplementary Table 1**. This was followed by a linear regression procedure to generate a standard curve for the metabolites of PAHs in urine. The limits of detection (LODs) for PAHs in urine samples ranged from 0.0023 to 0.0326 μg/L, while the spiked recoveries ranged from 68.19% to 107.05%. Values below the LOD were replaced by LOD/√2. Urinary creatinine (Cr) was quantified by the Department of Laboratory Medicine of the First Affiliated Hospital of Zunyi City (Model: AU680). The concentration of PAH metabolites in the urine of each pregnant woman was adjusted for the corresponding urine Cr concentration. Concentrations were expressed as µg/L Cr.

### Quality control

The standard addition method was used during the experiment: three different concentrations of standard solutions (low, medium, and high) were added into 1.5 mL urine, and each concentration of 8 tubes was parallel to calculate the recovery rate and precision. The limit of detection (LOD) was set up at a signal-to-noise ratio (S/N) of 3. For each batch of twenty urine samples, there was at least one experimental blank, and the relative deviation of two parallel samples should be less than or equal to 20% to deduct the inevitable background value for the experimental process and reagents. In addition, we monitored the intensity of the internal standard in each analysis, and the response value of the internal standard should be within ±30% of the response value of the calibration curve to avoid instrument bias or other interference. **Supplementary Table 2** displays the limits of detection, limits of quantification, recoveries, and precision of the PAH metabolism in our study.

### Definition and quantitative analysis of immune cells

The immunological parameters included in this study comprise: white blood cell count (×10^9^/L), lymphocyte count (×10^9^/L), neutrophil count (×10^9^/L), eosinophil count (×10^9^/L), basophil count (×10^9^/L), monocyte count (×10^9^/L), and platelet count (PLT, ×10^9^/L). All parameters were defined according to the Basic Principles and Methods for Interpretation of Clinical Laboratory Results (WS/T 402-2012) and the Guidelines for Approval of Blood Analysers and Related Tests (H20-A2). To ensure accuracy and reproducibility, a dual-quantification strategy was employed, combining routine clinical testing with internal laboratory validation. The specific workflow is as follows: Firstly, blood samples were collected from pregnant female subjects in a fasting, resting state, avoiding periods of uterine contraction. Vacuum blood collection tubes containing EDTA-K2 (1.8 mg EDTA-K2 per milliliter of blood) were used. Samples must undergo testing within one hour of collection. Should delayed testing be necessary, samples were stored at 4 °C (maximum 4 hours), followed by equilibration at room temperature for 30 minutes before analysis. Secondly, the XN-9000 fully automated blood analyzer, manufactured by Sysmex Corporation of Japan, was employed. This instrument utilizes a combined ‘electrical impedance + laser scattering’ detection technology to simultaneously perform white blood cell counts (including differential counts: neutrophils, lymphocytes, etc.) and platelet counts. Finally, manual verification is conducted using Wright-Giemsa staining. Under oil immersion microscopy, 200 white blood cells are counted to calculate differential percentages. These percentages are compared with analyze results, requiring >90% concordance (Kappa > 0.85). In case of discrepancy, manual results prevail. All results are synchronously entered into the Laboratory Information System (LIS), recording blood collection time, analysis time, instrument serial number, quality control batch number, and operator details. Data is linked to subject pregnancy information (gestational age, number of antenatal visits) to establish a complete traceability chain supporting subsequent data validation and repeatability verification.

Furthermore, relevant inflammatory markers were derived from previously published studies: Systemic Immune-Inflammation Index (SII) = platelets × neutrophils/lymphocytes; Aggregate Index of Systemic Inflammation (AISI) = platelets × neutrophils × monocytes/lymphocytes; neutrophil-to-lymphocyte ratio (NLR); platelet-to-lymphocyte ratio (PLR); and monocyte-to-lymphocyte ratio (MLR).

### Research variables

The independent variables in this study were obtained from laboratory data, dependent variables from the hospital system, and basic information about the pregnant women and their families were obtained from responses to a questionnaire. The independent variables included 1-OHNa, 2-OHNa, 2-OHFlu, 1-OHPh, 2-OHPh, 3OHPh, 4-OHPh, 9-OHPh,9-OHFlu, and 1-OHPYR. The dependent variables included maternal WBC, AISI, SII, NLR, PLR and MLR. Correction factors: This study used prior theory and literature review methods, and ultimately included covariates such as maternal age, pre pregnancy BMI, education level, household income, passive smoking, occupational exposure, etc. (24).

### Statistical analysis

The Kolmogorov–Smirnov test was used to assess data normality. Continuous variables were presented as mean ± standard deviation (s.d.) for normally distributed data and as median (interquartile range, IQR) for non-normally distributed variables; categorical variables were reported as percentages. PAH metabolite concentrations and Immune inflammatory indices were natural-log transformed prior to analysis to account for their right-skewed distribution. Covariates included maternal age, ethnicity, educational level, occupation, marital status, pre-pregnancy body mass index (BMI), smoking status, gravidity, spouse’s educational level, spouse’s occupation, annual household income, spouse’s smoking status, and gestational age. Analyses were conducted in five sequential steps: (1) pearson correlation analysis to assess associations between the raw concentrations of PAH metabolites and immune-inflammatory indices; (2) generalized linear regression models to examine associations between individual PAH metabolites and maternal immune-inflammatory indices; (3) Bayesian kernel machine regression (BKMR) to evaluate the combined effects of PAH metabolites mixtures on these indices; (4) restricted cubic splines (RCS) to explore dose–response relationships between PAH metabolites and immune-inflammatory indices; and (5) weighted quantile sum (WQS) regression to quantify the relative weight of each PAH metabolites in associations with these indices. All statistical analyses were performed using SPSS version 29.0 (IBM Corp., Armonk, NY, USA) and R version 4.3.0 (R Core Team; https://cran.r-project.org). The Benjamini Hochberg (BH) method was used for calibration in all analysis processes. Statistical significance is defined as the adjusted bilateral P<0.05.

## Results

### Characteristics of participants

A total of 2,581pregnant women were included in this study, the majority of whom were Han Chinese (98.0%). The age distribution was concentrated between the ages of 20 and 35, representing 94.0% of the total sample. The pre-pregnancy health status was average, with a pre-pregnancy BMI of 18.5-23.9 kg/m², accounting for 61.6%. Most participants had completed secondary school (80.0%). Most families were characterized by stable marital and income status. The study population exhibited a low prevalence of active smoking during pregnancy, yet a considerable proportion reported regular exposure to secondhand smoke. See **Table 1**.

**Table 1:** Baseline information about pregnant women.

Variable		N=2581 (%)
Age (years)	20–35 36–45	2388 (92.52%) 193 (7.48%)
Nation	Han Other	2530(98.02%) 51(1.98%)
Education	Low Middle High	84 (3.25%) 2067(80.09%) 430 (16.66%)
Employment status	Employed Unemployed	1495(57.92%) 1086 (42.08%)
Marriage status	Married Unmarried	2460 (95.31%) 121 (4.69%)
Pre-pregnancy BMI (kg/m^2^)	<18.5 18.5–23.9 >23.9	292 (11.31%) 1593 (61.72%) 696 (26.97%)
Husband’s education	Low Middle High	35(1.36%) 2079(80.55%) 467(18.09%)
Husband’s occupation	Employed Unemployed	2052(79.50%) 529(20.50%)
Annual household income	<100000 100000–200000 >200000	783 (30.34%) 1592 (61.68%) 206 7.98%)
Active smoking	Yes No	24 (0.93%) 2557 (99.07%)
Spouse’s smoking	Yes No	1094 (42.39%) 1487 (57.61%)
Gestational week	Mean ± SD	28.71 ± 14.24

Low: primary school education and below. Middle: junior high school education. High: college education and beyond.

### Concentrations of PAH metabolites in the urine of pregnant women

A total of 10 PAH metabolites were identified in the urine of pregnant women in this study, and their concentrations were described by median (95% CI) due to the skewed distribution of the data. The highest concentrations of 1-OHNAP and 2-OHNAP were observed at 0.36 (0.01, 1.46) µg/L Cr and 0.02 (0.00, 1.25) µg/L Cr, respectively. In contrast, the lowest concentrations of 4-OHPH and 9OHPH were noted at 0.04 (0.02, 0.11) µg/L Cr and 0.02 (0.00, 0.12) µg/L Cr, respectively, see **Table 2**.

**Table 2:** Concentrations of PAH metabolites in the urine of pregnant women (µg/L Cr).

Statistic	N	Mean	SD	Min	Max	Median	P25	P75	IQR	SE	95% CI
1-OHNAP	2581	2.73	15.99	0	479.53	0.36	0.01	1.46	1.45	0.32	2.12 - 3.35
2-OHNAP	2581	2.63	12.71	0	347.63	0.02	0.00	1.25	1.25	0.25	2.14 - 3.12
9-OHFLU	2581	1.96	30.42	0	1430.21	0.34	0.01	0.99	0.98	0.6	0.79 - 3.14
2-OHFLU	2581	1.73	29.55	0	1094.89	0.21	0.02	0.6	0.58	0.58	0.59 - 2.87
4-OHPH	2581	0.35	4.18	0	175.13	0.04	0.02	0.11	0.09	0.08	0.19 - 0.51
9-OHPH	2581	0.44	3.83	0	121.58	0.02	0.01	0.12	0.11	0.07	0.29 - 0.58
3-OHPH	2581	0.71	8.04	0	243.93	0.05	0.01	0.21	0.19	0.16	0.40 - 1.02
1-OHPH	2581	0.64	14.04	0	702.19	0.05	0.01	0.22	0.21	0.28	0.10 - 1.18
2-OHPH	2581	1.88	28.6	0	880.09	0.05	0.02	0.25	0.23	0.56	0.77 - 2.98
1-OHPYR	2581	0.44	6.51	0	322.66	0.02	0	0.13	0.13	0.13	0.18 - 0.69

### Levels of immune-inflammatory indices during pregnancy

This study analyzed six immunological inflammation indices. As the data exhibited a skewed distribution, the median (95% confidence interval) was employed for description. WBC count was 9.47 (7.98, 11.17)/L, AISI count was 436.08 (286.14, 671.77), SII count was908.90 (686.31, 1230.57), NLR count was 4.22 (3.33, 5.44), PLR was 129.22 (104.55, 160.00), and MLR was 0.29 (0.22, 0.37). See **Table 3** for details.

**Table 3:** Levels of immune-inflammatory indices during pregnancy.

Statistic	N	Mean	SD	Min	Max	Median	P25	P75	IQR	SE	95% CI
WBC	2581	9.73	2.43	3	21.9	9.47	7.98	11.17	3.19	0.05	9.64 - 9.82
SII	2581	1020.78	525.41	170.14	5385	908.9	686.41	1230.57	544.16	10.34	1000.50 - 1041.06
AISI	2581	549.64	437.7	14.07	6238.27	436.08	286.14	671.77	385.63	8.62	532.75 - 566.54
NLR	2581	4.74	2.3	0.79	40.25	4.22	3.33	5.44	2.11	0.04	4.65 - 4.83
PLR	2581	136.96	48.41	21.35	450	129.22	104.55	160	55.45	0.95	135.09 - 138.83
MLR	2581	0.32	0.15	0.05	1.62	0.29	0.22	0.37	0.15	0.00	0.31 - 0.33

### Association of PAH metabolites with immune-inflammatory indices

**Figure 1:**
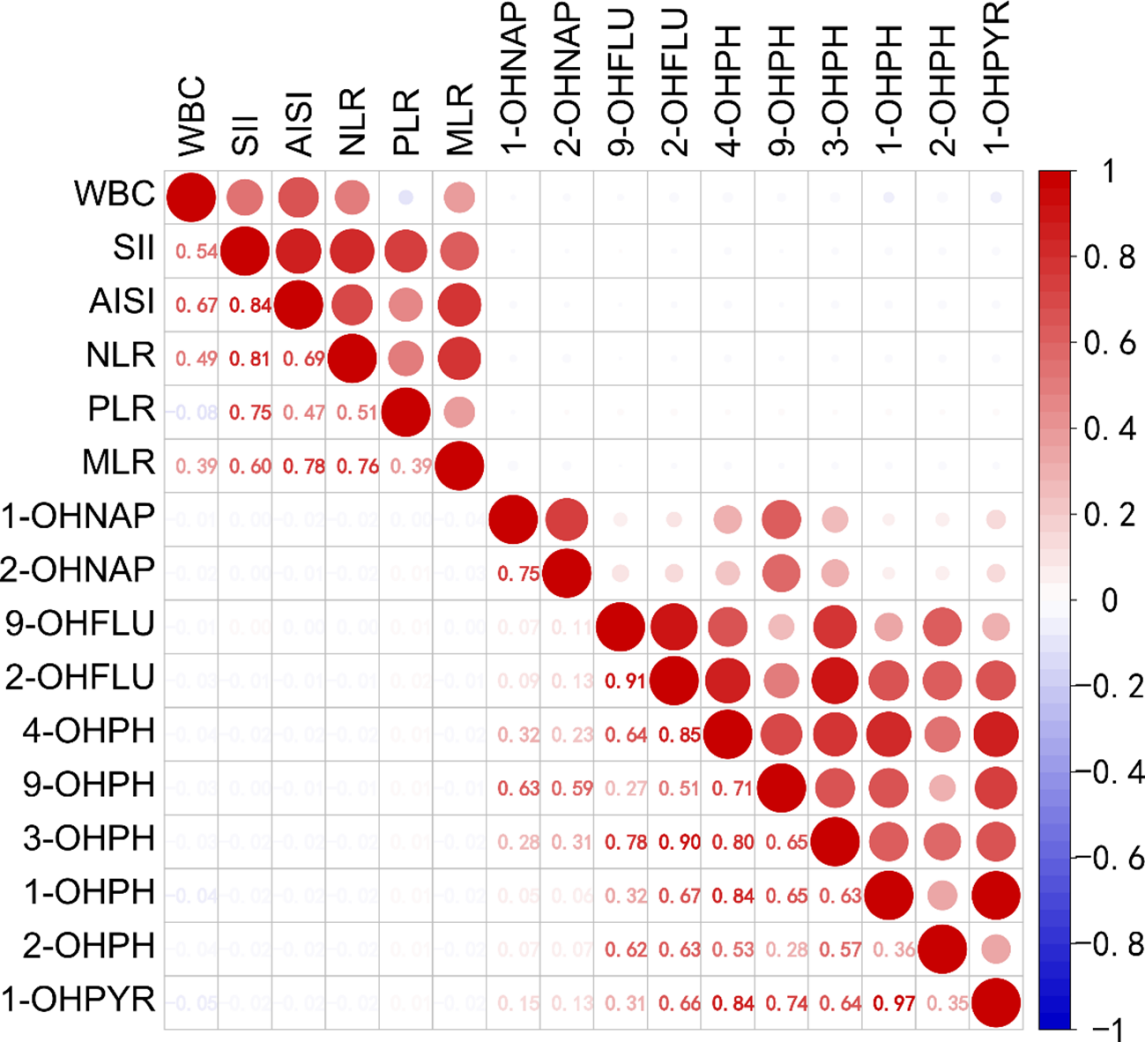
Association of PAH metabolites with immune-inflammatory indices. The redder the color, the stronger the positive correlation; The bluer the color, the stronger the negative correlation; White represents almost no correlation.

Here, we used Pearson correlation analysis to assess associations between the raw concentrations of PAH metabolites and immune-inflammatory indices. Our results showed moderate correlations (0.35 < r < 0.85) between white blood cell count (WBC), systemic immune-inflammation index (SII), aggregate index of systemic inflammation (AISI), neutrophil-to-lymphocyte ratio (NLR), platelet-to-lymphocyte ratio (PLR), and monocyte-to-lymphocyte ratio (MLR), with the exception of a weak correlation between WBC and PLR (r = −0.08). Among PAH metabolites, 9-hydroxyfluorene (9-OHFLU) and 2-hydroxyfluorene (2-OHFLU) were strongly correlated (r = 0.91); 9-hydroxyphenanthrene (9-OHPH) and 3-hydroxyphenanthrene (3-OHPH) also showed a strong correlation (r = 0.90); and 1-hydroxyphenanthrene (1-OHPH) and 1-hydroxypyrene (1-OHPYR) exhibited a very strong correlation (r = 0.97). With the exception of 1-hydroxynaphthalene (1-OHNAP) and 2-hydroxynaphthalene (2-OHNAP)—which showed weak correlations (r < 0.60) with other PAH metabolites—moderate correlations (0.40 < r < 0.80) were observed between all other PAH metabolites. Notably, WBC, SII, AISI, NLR, PLR, and MLR showed weak correlations (r < 0.1) with PAH metabolites (**Figure 1**).

### Association of PAH metabolites with maternal immune-inflammatory indices

**Figure 2:**
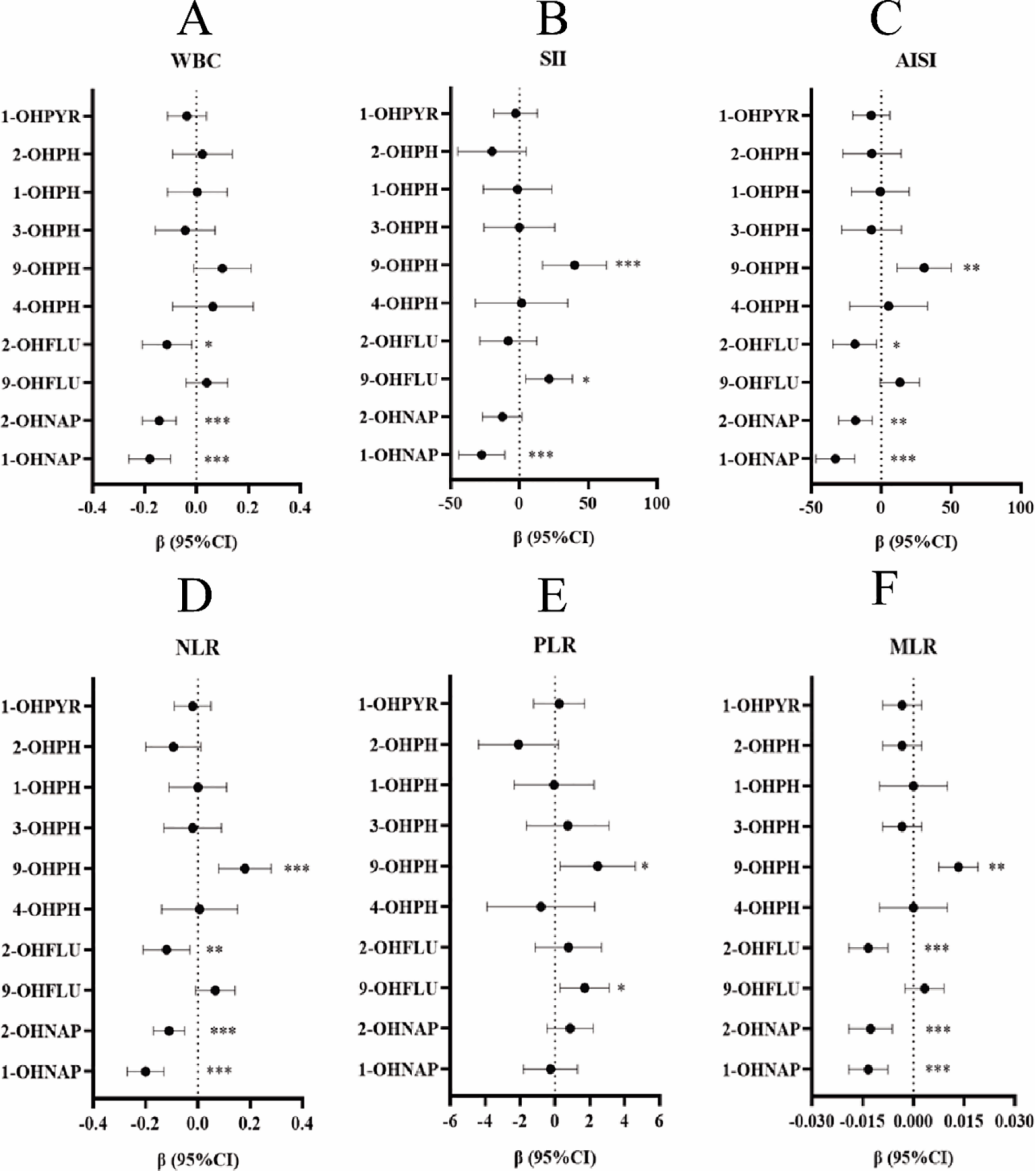
Association of PAH metabolites and maternal immune-inflammatory indices. Adjustment factors: Maternal age, ethnicity, educational attainment, occupation, marital status, pre-pregnancy BMI, smoking history, spouse's educational attainment, spouse's occupation, household annual income, spouse's smoking history, and gestational week. **(A)** shows the association between PAH metabolites and WBC. **(B)** shows the association between PAH metabolites and SII. **(C)** shows the association between PAH metabolites and AISI. **(D)** shows the association between PAH metabolites and NLR. **(E)** shows the association between PAH metabolites and PLR. **(F)** shows the association between PAH metabolites and MLR.

We used a generalized linear regression model to examine associations between PAH metabolites and maternal immune-inflammatory indices. Specifically, 1-hydroxynaphthalene (1-OHNAP), 2-hydroxynaphthalene (2-OHNAP), and 2-hydroxyfluorene (2-OHFLU) were associated with white blood cell (WBC) count, with β coefficients (95% confidence intervals [CIs]) of -0.18 (-0.26 to -0.10), -0.14 (-0.21 to -0.08), and -0.11 (-0.21 to -0.02), respectively. Additionally, 1-OHNAP, 9-hydroxyfluorene (9-OHFLU), and 9-hydroxyphenanthrene (9-OHPH) correlated with the systemic immune-inflammation index (SII), yielding β values (95% CIs) of -27.37 (-44.13 to -10.61), 21.60 (4.66 to 38.54), and 40.13 (16.79 to 63.46). For the aggregate index of systemic inflammation (AISI), 1-OHNAP, 2-OHNAP, 2-OHFLU, and 9-OHPH were associated with this marker, with β coefficients (95% CIs) of -32.78 (-46.71 to -18.85), -18.47 (-30.47 to -6.48), -17.85 (-35.02 to -0.67), and 30.86 (11.42 to 50.30). Furthermore, 1-OHNAP, 2-OHNAP, 2-OHFLU, and 9-OHPH correlated with the neutrophil-to-lymphocyte ratio (NLR), with β values (95% CIs) of -0.20 (-0.27 to -0.13), -0.11 (-0.17 to -0.05), -0.12 (-0.21 to -0.03), and 0.18 (0.08 to 0.28). 9-OHFLU and 9-OHPH were associated with the platelet-to-lymphocyte ratio (PLR), with β coefficients (95% CIs) of 1.61 (0.05 to 3.17) and 2.46 (0.31 to 4.62). Finally, 1-OHNAP, 2-OHNAP, 2-OHFLU, and 9-OHPH correlated with the monocyte-to-lymphocyte ratio (MLR), with β values (95% CIs) of -0.01 (-0.02 to -0.01), -0.01 (-0.02 to -0.01), -0.01 (-0.02 to -0.01), and 0.01 (0.01 to 0.02) (**Figure 2**).

### Non-linear relationship between PAH metabolites and maternal immune-inflammatory indices

**Figure 3:**
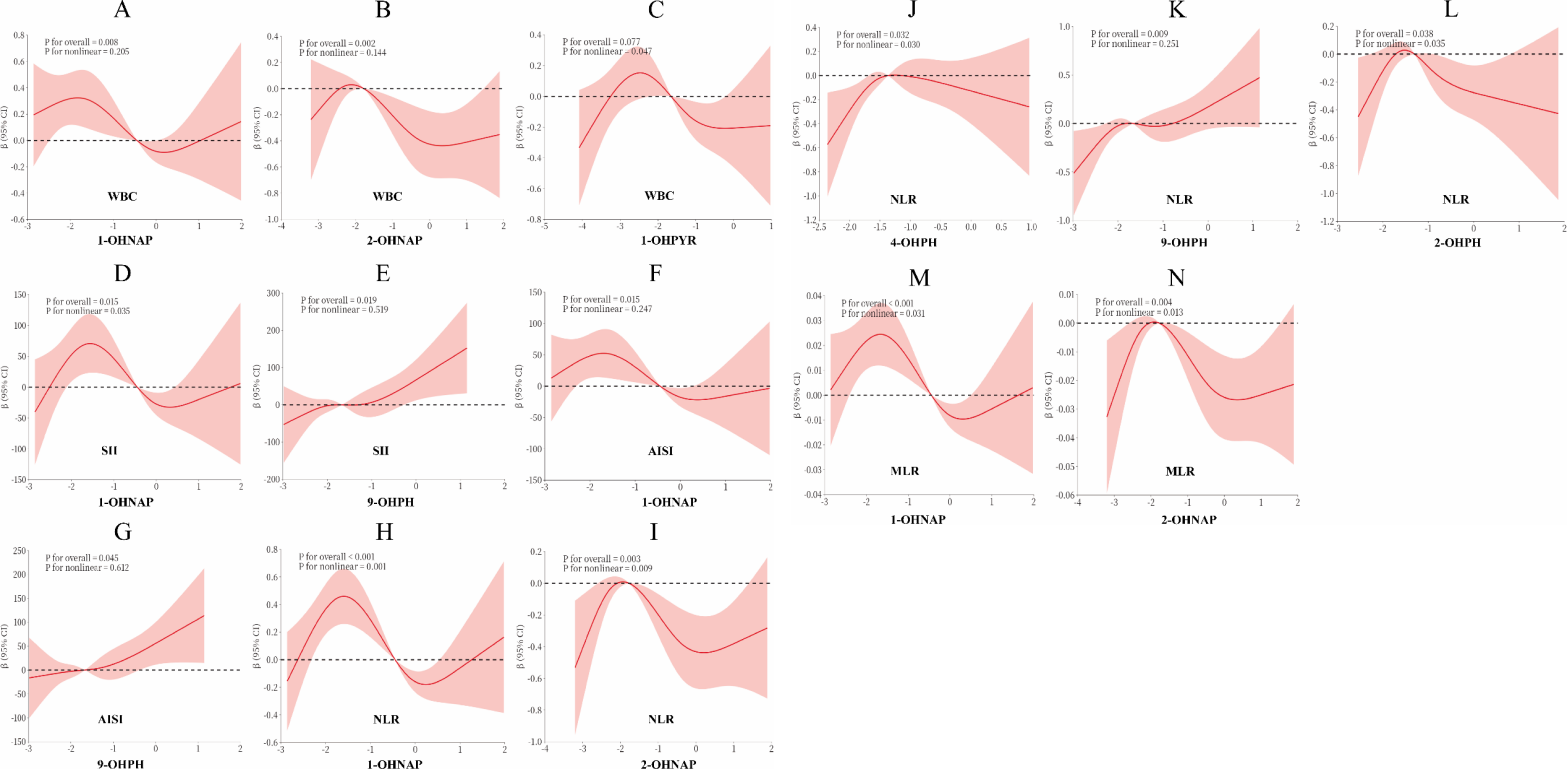
Non-linear relationship between PAH metabolites and maternal immune-inflammatory indices. Adjustment factors: Maternal age, ethnicity, educational attainment, occupation, marital status, pre-pregnancy BMI, smoking history, spouse's educational attainment, spouse's occupation, household annual income, spouse's smoking history, and gestational week. **(A–C)** illustrate the nonlinear correlation between PAH metabolites and WBC. **(D, E)** show the nonlinear correlation between PAH metabolites and SII. **(F, G)** shows the nonlinear correlation between PAH metabolites and AISI. **(H–L)** shows the nonlinear correlation between PAH metabolites and NLR. **(M, N)** shows the nonlinear correlation between PAH metabolites and MLR.

We used restricted cubic spline functions to further explore the relationship between polycyclic aromatic hydrocarbon (PAH) metabolites in maternal blood during pregnancy and immune-inflammatory indices. Specifically, 1-hydroxynaphthalene (1-OHNAP), 2-hydroxynaphthalene (2-OHNAP), and 1-hydroxypyrene (1-OHPYR) showed non-linear associations with white blood cell (WBC) count (overall P < 0.05, non-linear P < 0.05). Additionally, 1-OHNAP and 9-hydroxyphenanthrene (9-OHPH) exhibited non-linear relationships with the systemic immune-inflammation index (SII) (overall P < 0.05, non-linear P < 0.05), and the same two metabolites also demonstrated non-linear associations with the aggregate index of systemic inflammation (AISI) (overall P < 0.05, non-linear P < 0.05). Furthermore, 1-OHNAP, 2-OHNAP, 2-hydroxyphenanthrene (2-OHPH), 4-hydroxyphenanthrene (4-OHPH), and 9-OHPH showed non-linear relationships with the neutrophil-to-lymphocyte ratio (NLR) (overall P < 0.05, non-linear P < 0.05). Notably, 1-OHNAP and 2-OHNAP exhibited non-linear associations with the monocyte-to-lymphocyte ratio (MLR) (overall P < 0.05, non-linear P < 0.05). No non-linear associations were observed between PAH metabolites and the platelet-to-lymphocyte ratio (PLR) (**Figure 3**).

### Combined effects of PAH metabolites on maternal immune-inflammatory indices

**Figure 4:**
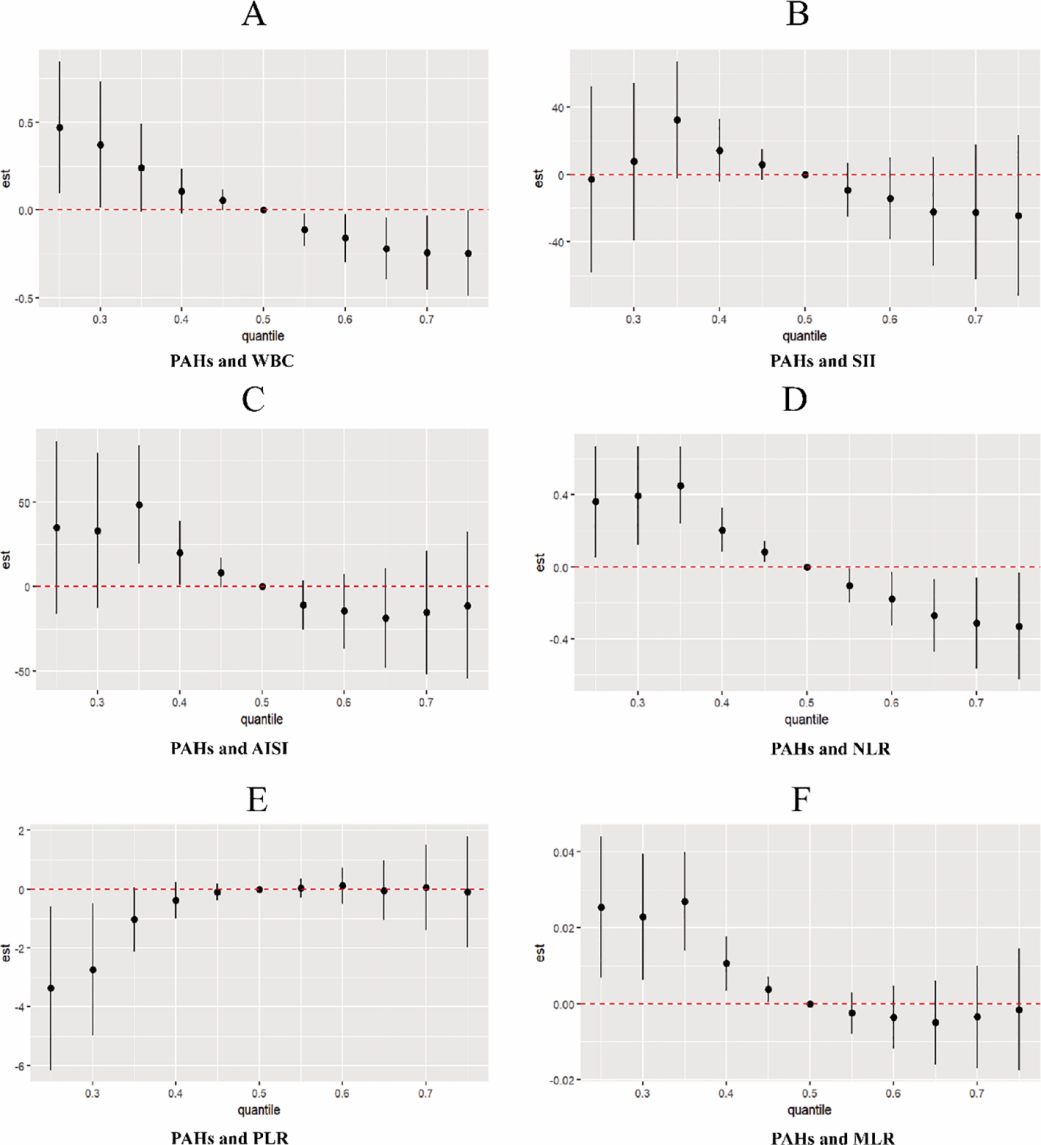
Combined effects of PAH metabolites on maternal immune-inflammatory indices (BKMR). Adjustment factors: Maternal age, ethnicity, educational attainment, occupation, marital status, pre-pregnancy BMI, smoking history, spouse's educational attainment, spouse's occupation, household annual income, spouse's smoking history, and gestational week. **(A)** shows the joint association between PAH metabolites and WBC. **(B)** shows the joint association between PAH metabolites and SII. **(C)** shows the joint association between PAH metabolites and AISI. **(D)** shows the joint association between PAH metabolites and NLR. **(E)** shows the joint association between PAH metabolites and PLR. **(F)** shows the joint association between PAH metabolites and MLR.

Given that polycyclic aromatic hydrocarbon (PAH) metabolites do not occur in isolation in the environment, and previous studies have largely focused on analyzing individual pollutants with limited exploration of their combined effects, we used the Bayesian kernel machine regression (BKMR) model to investigate the combined associations of PAH metabolites with immune-inflammatory indices. Our results indicate that PAH metabolites were negatively associated with total white blood cell (WBC) count (**Figure 4A**); were not significantly associated with the systemic immune-inflammation index (SII) overall (**Figure 4B**); showed no significant overall association with the aggregate index of systemic inflammation (AISI) (**Figure 4C**); were negatively associated with the neutrophil-to-lymphocyte ratio (NLR) overall (**Figure 4D**); were overall negatively associated with the monocyte-to-lymphocyte ratio (MLR) at low combined concentrations (**Figure 4E**); and exhibited an overall positive association with the platelet-to-lymphocyte ratio (PLR) at low combined concentrations (**Figure 4F**).

### Weighted analysis of PAH metabolites and maternal immune-inflammatory indices

**Figure 5:**
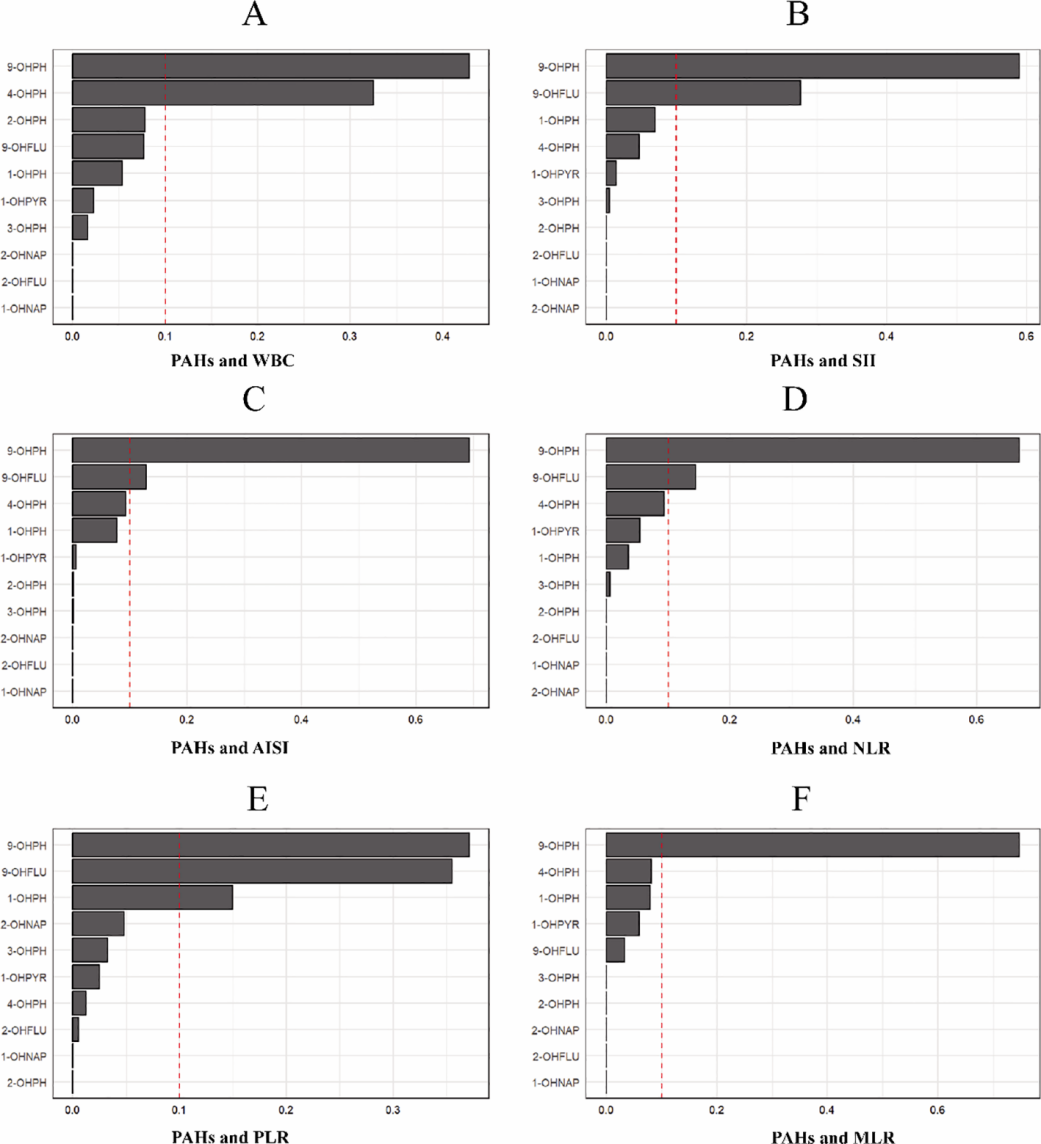
Combined effects of PAH metabolites on maternal immune-inflammatory indices (WQS). Adjustment factors: Maternal age, ethnicity, educational attainment, occupation, marital status, pre-pregnancy BMI, smoking history, spouse's educational attainment, spouse's occupation, household annual income, spouse's smoking history, and gestational week. **(A)** shows the weight analysis of PAH metabolites and WBC. **(B)** shows the weight analysis of PAH metabolites and SII. **(C)** shows the weight analysis of PAH metabolites and AISI. **(D)** shows the weight analysis of PAH metabolites and NLR. **(E)** shows the weight analysis of PAH metabolites and PLR. **(F)** shows the weight analysis of PAH metabolites and MLR.

We further used weighted quantile sum (WQS) regression to quantify the relative weights of individual polycyclic aromatic hydrocarbon (PAH) metabolites with respect to maternal immune-inflammatory markers. Our results showed that for white blood cell (WBC) count, the top three weighted PAH metabolites most strongly associated with this marker were 9-hydroxyphenanthrene (9-OHPH), 4-hydroxyphenanthrene (4-OHPH), and 2-hydroxyphenanthrene (2-OHPH); for the systemic immune-inflammation index (SII) — correcting the original “Inflammation Marker Index” to align with prior definitions — the top three were 9-hydroxyfluorene (9-OHFLU), 9-OHPH, and 1-hydroxyphenanthrene (1-OHPH); for the aggregate index of systemic inflammation (AISI), the top three were 9-OHFLU, 9-OHPH, and 4-OHPH; for the neutrophil-to-lymphocyte ratio (NLR), the top three were 9-OHFLU, 9-OHPH, and 4-OHPH; for the platelet-to-lymphocyte ratio (PLR), the top three were 9-OHFLU, 9-OHPH, and 1-OHPH; and for the monocyte-to-lymphocyte ratio (MLR), the top three were 9-OHPH, 4-OHPH, and 1-OHPH (**Figure 5**).

## Discussion

The recoveries of PAH metabolites in this study ranged from 80% to 110%, with standardized recoveries ranging from 80% to 120% (25). The main PAH metabolites found in the urine of pregnant women were 2-OHNAP and 1-OHNAP, a finding consistent with other studies (26). Secondly, the concentration range of PAHs in this study was 0.0007-1.4645 μg/L Cr, which is comparable to the Polish region (0.02-10.2 μg/L Cr) (27, 28), but lower than that of Guiyu (6.87 μg/L Cr) and Haojiang (3.90 μg/L Cr) (29). and Wuhan (0.74 µg/L C to 6.40 µg/L Cr) (30). This may be due to the limited number of exposure sources, low exposure doses, and diverse exposure spectra of PAHs in the environment of Southwest China (2, 31).

Our study demonstrates that exposure to polycyclic aromatic hydrocarbons (PAHs) during pregnancy is associated with maternal immune-inflammatory indices. Notably, the observed dose-response relationships and synergistic effects suggest that such exposure modulates maternal immune function. Early immunotoxicological studies have shown that multiple environmental chemicals can disrupt components of the rodent immune system. Over the past decades, growing attention has centered on the role of environmental pollutants in the increasing incidence and prevalence of immune-mediated diseases, including immunosuppression, allergic conditions, and autoimmune disorders. Mounting evidence supports the potential pathophysiological and immunotoxic effects of environmental pollutants in humans (32–36). Chronic exposure to PAHs adsorbed onto particulate matter has been linked to allergic diseases, immunodeficiency states, and autoimmune conditions (37–39).

Second, studies have shown that polycyclic aromatic hydrocarbons (PAHs) undergo enzymatic metabolism to convert from lipophilic to hydrophilic derivatives, which are then further detoxified into inactive molecules (32, 40). This metabolic process activates the aryl hydrocarbon receptor (AHR) pathway, thereby triggering an immune response. Consistent with this, animal studies have demonstrated that PAH exposure reduces thymic T lymphocyte counts in rats through activation of the AHR pathway (41–43). Furthermore, exposure to PAHs or other AHR ligands induces regulatory T (Treg) cell expansion, thereby promoting immunosuppression. This effect is mediated by multiple mechanisms, including direct transcriptional activation of target genes, epigenetic regulation of *Foxp3* transcription, and modulation of dendritic cell function (44–49). Consequently, PAH-associated autoimmune diseases may involve AHR-mediated induction of T helper 17 (TH17) cells — correcting the original “aromatase receptor” to align with prior AHR pathway definitions. Additionally, Hood et al. reported that PAHs may activate additional signaling pathways—including NF-κB and NFAT—to regulate immune function (50, 51). Collectively, these findings indicate that PAH metabolites are closely linked to immune system function.

### Strengths and weaknesses

Here, we employed multiple statistical approaches to characterize the associations between urinary PAH metabolites during pregnancy and maternal immune–inflammatory markers, and identified clear dose–response relationships and potential synergistic effects. Nevertheless, several limitations warrant consideration. First, as an observational study, causal inferences cannot be established. Second, we did not adjust for key potential confounders including maternal nutritional status and physical activity during pregnancy. Third, urinary PAH metabolites were measured only at a single time point, which may not fully capture exposure dynamics across gestation. Fourth, we used composite peripheral blood inflammatory indices—including the NLR, SII, and AISI—as surrogate markers of immune status. While these indices reflect systemic inflammatory burden, they do not directly quantify specific immune cell subsets, cytokine profiles, or detailed immune function. Accordingly, our findings support a potential link between PAH exposure and perturbations in maternal immune–inflammatory homeostasis. Future studies incorporating direct immune measurements—such as flow cytometry and targeted cytokine profiling—are needed to validate and extend these observations.

## Conclusion

In conclusion, this large birth cohort study demonstrates that prenatal exposure to PAHs is significantly associated with alterations in maternal immune-inflammatory indices. We observed clear dose–response relationships and synergistic combined effects of PAH metabolites on maternal immune profiles. Key contributing metabolites driving these associations include 9-OHFLU, 9-OHPH, 4-OHPH, 2-OHPH, and 1-OHPH. Our findings provide novel epidemiological evidence that prenatal PAH exposure may disrupt maternal immune homeostasis during pregnancy, supporting the importance of targeted interventions to reduce PAH exposure and protect maternal-infant health.

## Data Availability

The original contributions presented in the study are included in the article/**Supplementary Material**. Further inquiries can be directed to the corresponding authors.
