# Supplementary material for: Association between maternal PAH exposure and immune-inflammatory indices during pregnancy
Source: Front Immunol. 2026 Jun 9;17:1852582. doi: 10.3389/fimmu.2026.1852582 (PMC13286827; doi:10.3389/fimmu.2026.1852582)
Supplement: Supplementary file 1 [file Table1.docx]

| Table S1 On-board specific information table for phthalate metabolites | | | | |
| --- | --- | --- | --- | --- |
| metabolite | retention time | parent ion | daughter ion | collision energy |
| 1-OHNAP | 16.043 | 216 | 201 | 15 |
| 2-OHNAP | 16.547 | 216 | 201 | 15 |
| 9-OHFLE | 20.231 | 254 | 165 | 20 |
| 2-OHFLE | 22.471 | 254 | 239 | 15 |
| 4-OHPHE | 25.209 | 266 | 235 | 25 |
| 9-OHPHE | 25.211 | 266 | 73 | 25 |
| 1-OHPHE | 25.216 | 266 | 73 | 25 |
| 3-OHPHE | 25.854 | 266 | 73 | 25 |
| 2-OHPHE | 26.702 | 266 | 73 | 25 |
| 1-OHPYR | 30.857 | 290 | 73 | 25 |

Table S2 Limit of detection (LOD), limit of quantification (LOQ), recovery rate, and precision.

| compound | internal standard | regression equation | Decision coefficient (R^2^) | LOD (ug/L) | LOQ (ug/L) | recovery rate（R%） | precision（%） |
| --- | --- | --- | --- | --- | --- | --- | --- |
| 1-OHNAP | 1-OHNAP-D7 | y=0.0367x+0.0126 | 0.9998 | 0.0050 | 0.0167 | 107.05 | 1.40 |
| 2-OHNAP | 1-OHNAP-D7 | Y=0.0509x+0.0035 | 0.9999 | 0.0027 | 0.0090 | 105.71 | 2.27 |
| 9-OHFLE | 1-OHNAP-D7 | Y=0.0371x-0.0011 | 0.9998 | 0.0023 | 0.0078 | 94.15 | 2.85 |
| 2-OHFLE | 1-OHNAP-D7 | Y=0.0663x-0.0013 | 0.9998 | 0.0029 | 0.0098 | 91.99 | 3.18 |
| 4-OHPHE | 1-OHNAP-D7 | Y=0.0409x+6.1544 | 0.9999 | 0.0167 | 0.0556 | 90.08 | 5.01 |
| 9-OHPHE | 1-OHNAP-D7 | Y=0.2914x-0.0055 | 0.9982 | 0.0044 | 0.0147 | 105.01 | 4.99 |
| 1-OHPHE | 1-OHNAP-D7 | Y=0.0383x-0.0016 | 0.9998 | 0.0071 | 0.0236 | 81.09 | 4.96 |
| 3-OHPHE | 1-OHNAP-D7 | Y=0.0188x-0.0010 | 0.9999 | 0.0095 | 0.0316 | 84.50 | 5.48 |
| 2-OHPHE | 1-OHNAP-D7 | Y=0.0171x-4.7902 | 0.9999 | 0.0115 | 0.0385 | 68.19 | 2.41 |
| 1-OHPYR | 1-OHPYR-D9 | Y=1.770x+0.0213 | 0.9999 | 0.0003 | 0.0011 | 100.30 | 1.97 |
